# Psychological Well-Being and Self-Efficacy for Self-Regulated Learning

**DOI:** 10.3390/ijerph21081037

**Published:** 2024-08-07

**Authors:** Maria Luisa Pedditzi, Laura Francesca Scalas

**Affiliations:** Department of Education, Psychology, Philosophy, Faculty of Humanistic Studies, University of Cagliari, 09123 Cagliari, Italy; lfscalas@unica.it

**Keywords:** well-being, self-efficacy, self-regulated learning, adolescence

## Abstract

This study explores psychological well-being in adolescence through a multidimensional perspective using the Adolescent Students’ Basic Psychological Needs at School Scale, derived from the Self-Determination Theory. The ASBPNSS focuses on three basic psychological needs (Competence, Autonomy, and Relatedness) in adolescence and has not yet been used within the school context in Italy. This study’s main objectives are: (1) to validate a preliminary Italian version of the ASBPNSS; (2) to analyze the association between well-being at school and self-efficacy for self-regulated learning; and (3) to verify whether there are differences by gender. A sample of 395 students (mean age = 17.5; SD = 0.75) completed the ASBPNSS and the Self-Efficacy for Self-Regulated Learning Scale. The factorial structure, composite reliability, and gender invariance of the ASBPNSS were examined. Associations between well-being at school and self-efficacy were tested with structural equation models (CFI = 0.935, TLI = 0.925; RMSEA = 0.054). Measures of well-being were associated with school self-efficacy for self-regulated learning, which predicted Competence (beta = 0.639), Relatedness (beta = 0.350), and Autonomy (beta = 0.309). These relationships were invariant over gender, although girls reported lower latent means in the Relatedness factor. This study highlights the importance of promoting school self-efficacy and well-being in adolescence.

## 1. Introduction

There is no agreed definition of well-being [1]. The OECD’s—Organization for Economic Cooperation and Development [2]—definition of well-being includes factors such as health, education, salary, quality of accommodation, and quality of relationships. This approach defines well-being mainly in objective terms and does not consider psychological well-being. Psychological well-being is instead mainly a perceived psychological condition. Although well-being is influenced by objective conditions, well-being is primarily a psychological variable, and it is how well a person feels their life to be going [1]. There are two traditions in the study of psychological well-being: the hedonic approach and the eudaimonic approach [3]. The hedonic perspective is more related to the concept of life satisfaction and happiness [4,5] and it conceptualizes well-being mainly as pleasure or the absence of a negative mood [4,5]. The hedonic approach includes an affective component (positive and negative affect) and a cognitive component, which is often identified as life satisfaction [3,5,6]. On the other hand, the eudaimonic tradition insists that the essence of well-being is based on perceived functional ability, sense of meaning, and positive relationships [6,7,8,9]. This perspective describes well-being in terms of being fully functional as individuals and considers some specific dimensions of psychological well-being [6,7,8,9,10]. For Deci and Ryan [3,7,8], the key dimensions are Autonomy, Competence, and Relatedness, which they describe as basic psychological needs. Self-Determination Theory [11,12,13,14] is, therefore, an eudaimonic conceptualization of well-being because it assumes that well-being is the outcome of the satisfaction of three psychological needs: (1) Competence refers to the need to be able to achieve a good outcome, to feel that one is capable when doing something; (2) Autonomy refers the need to act, feel, and think freely; and (3) Relatedness captures the need to feel connected to others, and the need to be part of a community or a social group. When people do not meet these basic psychological needs they experience conflict and distress. 

The Basic Psychological Needs Theory—BPNT [7,8,12,13,14]—has generated a significant amount of research in several areas, such as work [15,16], sport [17,18], and education [19,20], but the research in the school context using specific measures for students is still limited.

### 1.1. Basic Psychological Need Satisfaction at School

Basic Psychological Needs Theory—BPNT [7,8,12,13,14]—is applicable to the environment of education because it offers a framework for understanding how contextual school variables can promote psychological well-being and academic achievement in students [21]. Well-being, frequently called wellness in the Self-Determination Theory literature [14], is typical of the eudaimonic approaches, focusing on the full functionality concept of well-being, related to self-awareness and self-regulated behaviors. This is particularly important in the school context. The main focus of this approach is on a healthy functioning self, involving the integrated processes of the autonomous, competent, and social functioning of a person [14]. Deci and Ryan [13] asserted that situational contexts that frustrate the satisfaction of the needs reduce well-being. On the contrary, factors such as educational opportunities and positive relations in the school context promote full functioning and, therefore, well-being [21,22].

Different studies have shown that basic need satisfaction promotes healthy psychological development, school motivation, and better academic performance in students [21,22]. The frustration of the three basic psychological needs was significantly associated with lower levels of positive mental health [23]. Studies about the influence of gender differences on the relationship between the basic psychological needs and mental health in adolescence [10,14,23] highlighted that higher levels of Relatedness frustration and Autonomy frustration predicted worse levels of mental health and the effects were stronger for girls than for boys. Previous studies conducted in the school context have found that, during adolescence, girls pay more attention to interpersonal relationships. In contrast, boys focus more on developing personal abilities and competence [24]. Thus, these differences between male and female adolescents may influence their perceptions of the basic psychological needs and social support, as well as the frustration of these needs at school. 

Research on basic psychological needs in the specific context of education is particularly relevant since it can help identify the factors (for example, peer relationships at school and teachers’ educational styles) that can improve and sustain students’ basic needs.

However, research on the basic needs in adolescents’ education is still limited, particularly, because it is based on general measures of basic need satisfaction, which do not consider differences across contexts and the specificity of students’ school experiences [25,26]. Since schooling represents one of the major life contexts of adolescents, Tian, Han and Huebner [25] proposed a specific construct “adolescent students’ basic psychological needs at school” and created a new measure based on the framework of BPNT [13,14]. This new construct can facilitate cross-cultural research about BPNT at school in different countries. The ASBPNSS [25] is an instrument that includes three different scales: Autonomy, Competence, and Relatedness. The need for Competence refers to students’ desires to develop and express their capabilities in the school activities. The need for Autonomy refers to students’ desires to experience a sense of freedom and self-determination of their behavior at school. The need for Relatedness refers to students’ desires to experience a sense of connection with classmates and teachers and a sense of belonging to the school. Tian et al. [25], using several samples of Chinese adolescent students, found evidence for the reliability and validity of the ASBPNSS. The findings of exploratory and confirmatory factor analysis procedures revealed a three-factor structure, consistent with the widely accepted, underlying theoretical model of Ryan and Deci [13,14]. The results showed acceptable internal consistency, reliability, and meaningful test–retest reliability for the scale [25]. Moreover, evidence of convergent and divergent validity was obtained, as well as evidence of predictive validity. Subsequently, psychometric assessments of translated versions [26,27,28], Persian and Turkish, were conducted. Recently, a Portuguese version has been developed [26]. However, despite its promising psychometric properties, the ASBPNSS has not been widely used for research outside of the Chinese context. Given this gap in the literature, we aim to develop an Italian version of the instrument and replicate research in the school context.

### 1.2. Self-Efficacy for Self-Regulated Learning and Psychological Well Being

Social cognitive theory focuses on the construct of perceived self-efficacy [29,30,31]. Perceived self-efficacy is concerned with people’s beliefs in their capability to produce a given level of attainment [29,30]. People with high self-efficacy set stimulating goals for themselves and monitor how to reach them and overcome obstacles [30,31]. Different research findings show that efficacy beliefs exert an impact on human development and adaptation [31,32,33]. The sources of self-efficacy vary according to contextual factors such as culture, ethnicity, gender, and ability domains [30,31], which includes the active mastery experience, vicarious experience, social persuasion or encouragement from others, and the physiological and affective states [32,33,34]. People vary in the areas of life in which they promote their sense of efficacy [35,36], because self-efficacy is not a global quality, but a discriminated set of beliefs in a particular domain of functioning [37,38]. 

In the school context, self-efficacy for self-regulated learning (SESRL) refers to the beliefs that students develop about their ability to use self-regulated learning (SRL) strategies to improve their learning and reach their goals [39]. Self-regulation is a metacognitive process that permits one to explore personal thought processes to evaluate actions and plan pathways to success [39,40]. Self-regulated learning is an active process, for monitoring, regulating, and controlling cognitions and actions based on the achievement goals and the learning environment [40,41]. According to social cognitive theory, people are proactive and self-regulating agents of their psychosocial development [29,30]. The self-regulation of action reflects the active control people exert by setting goals. Mastery is progressively achieved through the perception of causal relations between events and through the recognition of oneself as the agent of action [29].

Students’ beliefs in their efficacy to regulate their own learning activities affect their school motivation, academic achievement, and scholastic aptitude [32,34,35,36]. Self-efficacy for self-regulated learning [33,37] concerns students’ beliefs to use cognitive strategies, to plan academic activities, and to complete scholastic tasks. In Bandura’s Perceived Self-Efficacy Scale [37], perceived efficacy for self-regulated learning [33,37] measures students’ efficacy in organizing environments for learning, pursuing academic goals when there are other interesting things to do, using cognitive strategies, and motivating themselves to complete school tasks [33,37]. 

Social cognitive theory highlights sources and mechanisms through which self-efficacy beliefs affect behavior [38,39,40]; however, it is not clear yet how self-efficacy for self-regulated learning affects the well-being associated with basic psychological needs. 

Several studies have focused on the relationship between general self-efficacy and well-being in students [39,40]. From these studies, it has emerged that self-efficacy beliefs affect positive thinking and positive expectations [40]. Students with high-level self-efficacy are interested in well-being-related stimuli [40], and high self-efficacy also contributes to well-being and school engagement. 

Other studies have analyzed the relationship between perceived self-efficacy and well-being in terms of basic psychological needs in different fields, such as in sporting activities [42] and school activities [43], and have found correlations between perceived self-efficacy and the need for Competence [42,43]. In particular, in school activities, academic self-efficacy mediated the relationship between both Competence and Relatedness satisfaction, and learning engagement [43]. Furthermore, mastery experiences (Competence) and positive feedback (Relatedness) were the primary sources of self-efficacy [42]. However, these findings have not been replicated in a school setting using specific measures for basic psychological needs and self-efficacy in self-regulated learning. 

There is a gap in the literature regarding studies using the ASBPNSS [25] in association with the Perceived Efficacy for Self-Regulated Learning Scale [36,37]. Therefore, in accordance with Inman, Costa, and Moreira [26], we believe it is important to extend the research in this area to different contexts. There is also a need to include replications in cultural adaptations of the ASBPNSS in order to provide additional support for its invariance across gender [25].

### 1.3. Aims

Referring to the above-mentioned literature, we, first of all, carried out a preliminary validation of the ASBPNSS in the Italian school context. Another research objective of this study was to analyze the associations between the three dimensions of the Well-Being Basic Psychological Needs at School Scale and the Perceived Self-Efficacy for Self-Regulated Learning Scale. 

Specifically, (1) we hypothesized that self-efficacy for self-regulated learning would be associated to all measures of psychological well-being at school, but that it would more strongly affect the basic need for Competence at school, since they are theoretically connected [42,44,45]. Also, (2) we expected that the pattern of association between the constructs would be similar in males and females [25,26]. However, since previous studies conducted on adolescents [10,14,23,24] showed that girls reported lower levels of Relatedness than males, (3) we expected potential gender differences in latent means on the Relatedness basic need. 

## 2. Materials and Methods

### 2.1. Participants and Study Design 

The study design of this research is correlational. A sample of 395 students was involved in this study. Our participants were recruited through nonprobability sampling across secondary public schools (high schools) in Sardinia (Italy). Our sample is composed of students from Lyceum (70%.) and technical institutes (30%). Both of these routes are very common in Italy. The Italian secondary education system is divided into two stages and it lasts 8 years: lower secondary school or middle school (ages 11–14) and upper secondary school or high school (ages 14–19). There are three types of high school: Lyceum (which aims to prepare students for university); technical institutes (which aims to prepare both for work and for university) and institutes for specific professions (which includes practical work related to a specific industry). 

This study was approved by the Ethics Committee of the University of Cagliari. After approval from the school principals, informed consent was collected from the students or their parents for underage students. The survey was administrated via Google Forms during class, in the presence of one teacher and/or one research assistant. Nine control questions (e.g., to this item please respond 1) were included in the survey. A total of 447 students completed the ASBPNSS [25] and a self-efficacy scale [36]. Answers from 52 students were excluded since they failed one or more of the control questions. The final sample comprised 395 students attending the 2 final years of high school (F = 48.4%; M_age_ = 17.5, SD_age_ = 0.75). 

### 2.2. Measurements

A survey with Google Forms was implemented and administered online through a link in a work session of about 30 min. The first part of the protocol assessed the demographic variables (age, gender, and school). Then, the ASBPNSS of Tian, Han, and Huebner [25] and the Perceived Self-Efficacy for Self-Regulated Learning Scale of Pastorelli and Picconi [36] were used.

The ASBPNSS analyzed 3 dimensions: Autonomy, Competence, and Relatedness. Autonomy items measure the student’s desire to express themselves at school (for example: “I can decide for myself how to do things at school”). Competence items examine the student’s knowledge of school-related skills and a sense of effectiveness (for example: “I have been able to learn interesting new skills at school recently”). Relatedness items focus on the student’s desire to establish good relationships with classmates and teachers (for example: “Teachers and classmates are pretty friendly towards me at school”). Tian and colleagues [25] confirmed the three-dimensional a priori structure of the instrument with exploratory and confirmatory factor analysis; the factorial structure was found to be invariant across gender and partially invariant over age, and reliability (Cronbach’s alpha range: 0.77–0.85; Guttman split-half range: 0.61–0.77) was supported as well.

Since an Italian version of the instrument was not available, we translated and validated the scale (see Results section) and performed an initial validation of the instrument. We used a back-translation process with independent translators fluent in both Italian and English [44]. The instrument was translated by two independent experts and, after discussion on small disagreements, the final version was back-translated and evaluated by a third independent bilingual expert to make final adjustments [45]. The comprehensibility of the items was checked with a small group of students who did not report any difficulties. 

Although Tian and colleagues [25] organized the items of the ASBPNSS after factor analysis (thus having items tapping the same construct grouped), we preferred to randomly reorganize the items to alternate the three constructs. Therefore, we provide a double number for each item, the first number refers to Tian et al.’s item number after factorialization and the second number refers to the order of presentation in our study. 

As a measure of school self-efficacy, we used items from the Perceived Self-Efficacy Scale validated in Italian by Pastorelli and Picconi [36] and derived from the Children’s Perceived Self-Efficacy scales [35,37]. Specifically, we used 10 items referring to perceived efficacy in regulating own motivation and learning activities [38] of the Perceived Efficacy for Self-Regulated Learning Scale (items like: “How capable are you of finishing your homework assignments on time” or “How capable are you of committing yourself to studying when you have other interesting things to do”). The omega McDonald’s index computed in our sample was 0.986, thus showing the high reliability of the scale [46].

### 2.3. Statistical Analyses

As a preliminary step, we examined the factor structure and invariance of the Italian version of the ASBPNSS [25], since the instrument has not been validated in Italian yet. We used Confirmatory Factor Analysis (CFA) and Exploratory Structural Equation Modeling (ESEM) [47,48] procedures to examine the factorial structure of the scale. The CFA’s no-cross-loading requirement often brings inflated correlations among the latent factors in multidimensional instruments, such as the ASBPNSS, due to small correlations between items and non-target factors [49]. ESEM can overcome this issue with target rotation [48], which allows model specification in a confirmatory way, “targeting” all freely estimated cross-loadings to be close to 0, but allowing cross-loadings to avoid biased estimates.

We used McDonald’s [46] omega (ω) coefficient to evaluate reliability: ω = (Σ|λi|)^2^/([Σ|λi|]^2^ + Σδii) where λi is the factor loadings and δii is the error variance. 

We performed tests of measurement invariance across gender for the Italian version of the ASBPNSS [49]. As a first step, we tested configural invariance; as a second step, we moved to weak invariance (i.e., invariance of the factor loadings); as a third step, we examined strong invariance (i.e., invariance of factor loadings and intercepts); as a fourth step, we tested the invariance of uniquenesses, and then the invariance of variances and covariances; and finally, we evaluated the invariance of latent means.

To evaluate the effect of self-efficacy on the school’s basic needs measured by ASBPNSS, we applied structural equation models and we verified if the pattern of associations was invariant over gender. In an additional model, we also examined the latent interaction of gender with self-efficacy on the three dimensions of well-being at school. 

We used the robust maximum likelihood estimator and full information maximum likelihood [50] to deal with missing values (0.5%); therefore, in presenting our results, we also included the scaling correction factor (Scf). As suggested in the literature, we used several fit indices to evaluate the model’s [51] Chi-square test of exact fit (χ^2^), Comparative Fit Index (CFI), Tucker–Lewis Index (TLI), and Root Mean Square Error of Approximation (RMSEA). Concerning CFI and TLI, values higher than 0.90 suggest an adequate fit, whereas values higher than 0.95 imply an excellent fit to the data. RMSEA values smaller than 0.08 indicate acceptable fit and values smaller than 0.06 imply excellent model fit to the data [52]. Changes in CFI and RMSEA fit indices [51] were used to evaluate invariance tests, since differences in χ^2^ are oversensitive to sample size and minor misspecifications. A decline of 0.01 or less for CFI and an increase of 0.015 or less for RMSEA indicates that the more parsimonious (i.e., invariant) model should be retained. Data analyses were conducted using the software Mplus 7.3 [52] and SPSS and Amos graphics version 22.0 [53]. 

## 3. Results

*Italian validation of the ASBPNSS*. To examine the factorial structure of the ASBPNSS, we used both CFA and ESEM (Table 1). The CFA model based on all items showed poor factor loadings, non-significant (FL = 0.049 S.E. = 0.062) or trivial (FL = 0.172 S.E = 0.070) factor loadings for two items (respectively, item 10_14 and item 15_15), and double saturation for two additional items (item 2_4 and item 5_13). Latent correlations among the factors were between 0.62–0.73. ESEM showed sufficient fit indices and, as expected, lower latent factors (range between 0.52–0.62) due to the absence of the zero-factor-loading constraints on non-target factors. Nonetheless, ESEM confirmed results similar to the CFA in relation to trivial saturations for items 10_14 and 15_15, and double saturations for items 2_4 and 5_13. It should be noted that items 10_14 and 2_4 were also problematic in the validation study. Indeed, Tian and colleagues reported double saturations for these two items [25].

The elimination of these four items improved fit indices in both CFA and ESEM, with closer values in terms of factor loadings (respectively, 0.58–0.93 and 0.36–0.92, see Table 2), uniquenesses (respectively, 0.58–0.93 and 0.36–0.92), and latent correlations (respectively, 0.53–0.70 and 0.48–0.63, see Table 3). Also, omega reliability indices were similar in CFA and ESEM (see Table 2).

**Figure 1:**
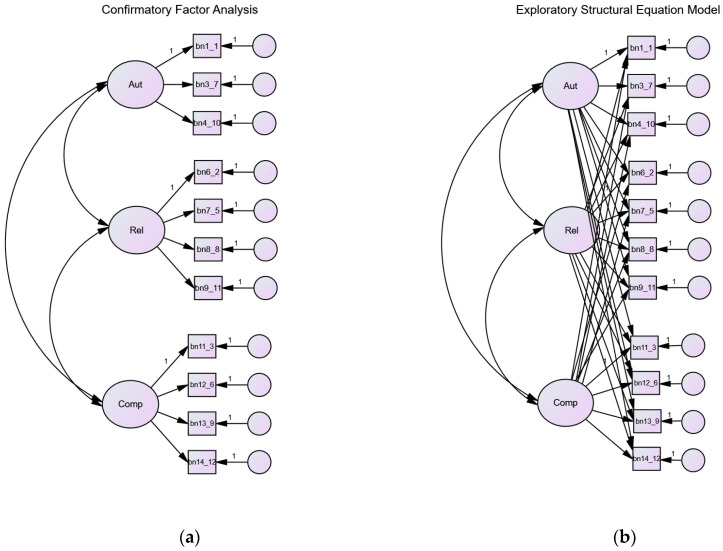
Measurement models of the ASBPNSS with the 11 items examined in this study: (**a**) Confirmatory Factor Analysis; (**b**) Exploratory Structural Equation Model.

Since the ESEM solution for the 11-item version of the scale did not provide substantially different estimates compared to the CFA, subsequent analyses were based on the more parsimonious CFA model. The 11-item CFA and ESEM can be observed in Figure 1. As found in the previous literature [25,26], the 11-item CFA model was completely invariant over genders (see Table 1), with only one difference between males and females for the Relatedness latent mean, which was lower in females (−0.299, S.E. = 0.076).

**Figure 2:**
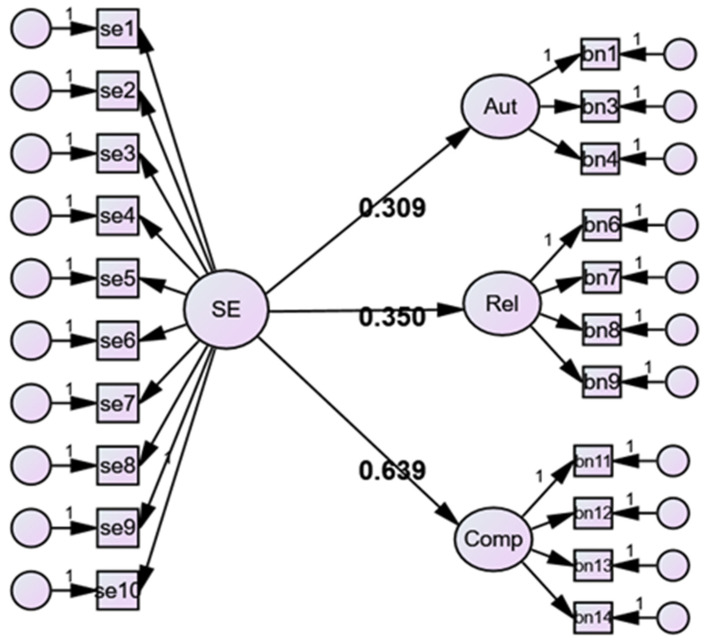
Effects of Regulatory Self-efficacy on Autonomy, Relatedness, and Competence School Basic Needs.

*Effects of regulatory self-efficacy at school on psychological well-being*. Using structural equation models, we tested the effects of regulatory self-efficacy at school on Autonomy, Competence, and Relatedness basic needs (Figure 2). Based on the previous literature [39,40], we hypothesized that regulatory self-efficacy would affect all three dimensions of school basic needs, especially Competence basic needs [42,44,45]. The fit of the model was adequate (χ^2^ = 392.273, df = 181, Scf = 1.11, CFI = 0.935, TLI = 0.925, RMSEA 0.054). Regulatory self-efficacy affected all factor of well-being at school (Autonomy = 0.309, S.E = 0.062; Competence = 0.639, S.E. = 0.057; Relatedness = 0.350, S.E. = 0.058) and, in particular, better explained Competence (r-square = 0.409, S.E. = 0.073); it also contributed to the explanation of Relatedness (r-square = 0.122, S.E. = 0.040), although, to a lesser extent, Autonomy was weaker (r-square = 0.096, S.E. = 0.038).

We tested if the pattern of associations between the three basic needs and regulatory self-efficacy was invariant over gender (Table 4). Previous studies [23,24] showed that girls reported lower levels of Relatedness satisfaction in adolescence than males. Therefore, we expected potential differences in latent means on the Relatedness basic need.

The analyses revealed invariant measurement structure, betas, and variances/covariances. Only differences in the latent mean of the Relatedness factor emerged, as expected, with females less satisfied with their relations at school.

Finally, using the LMS method implemented in Mplus, we explored the effect of latent interaction of regulatory academic self-efficacy with gender on school basic needs; all the effects of latent interaction were not statistically significant (unstandardized betas, Autonomy = −0.120, S.E. = 0.137, Competence = −0.053, S.E. = 0.125, and Relatedness = 0.024, S.E. = 0.141).

## 4. Discussion

This study attempted to investigate which dimensions of the ASBPNSS [25] might be related to self-efficacy for self-regulated learning. Since the ASBPNSS has not been validated in Italian yet, we proposed an initial validation of the scale. The initial 15-item version presented some flaws, as shown by the comparison between the CFA and ESEM. Specifically, two items showed double saturations and two additional items had trivial and non-significant saturations on the expected factors. After eliminating these items, the scale’s psychometric properties improved substantially and showed invariance over gender. 

The findings underlined a connection between self-efficacy for self-regulated learning and basic psychological needs, specifically with the psychological need for competence at school. These results confirm previous research data about the relationship between self-efficacy and well-being in adolescence [40,42]. However, our results show more about the relationship between self-efficacy for self-regulated learning and well-being in adolescence, particularly about self-efficacy for self-regulated learning and basic psychological needs. Furthermore, we found that, as we hypothesized, self-efficacy for self-regulated learning more strongly affects the basic need for competence at school, as already found in the previous literature [42,43]. Also, as we hypothesized, the pattern of association between the constructs was similar in males and females [25,26]. The gender difference that emerged was for the latent mean of Relatedness, which was significantly lower for females. Previous studies about the influence of gender differences on the relationship between basic psychological needs and mental health in adolescence [10,14,23,24] had highlighted that higher levels of Relatedness predicted worse levels of mental health and that the effects were stronger for girls than for boys. Previous studies conducted in the school context have found that, during adolescence, girls pay more attention to interpersonal relationships [24]. Thus, these differences between male and female adolescents may influence their perceptions of the basic psychological needs and social support, as well as the frustration of these needs at school. 

Furthermore, studies on satisfaction in relationships with peers in adolescence indicate that the satisfaction expressed by adolescents varies by gender, age, and context [54,55,56], and depends on the quality of relationships in the classroom [57,58]. Studies conducted in Italian high schools [55,56,59,60] have highlighted that boys seem to be more satisfied than girls in their relationships with classmates; contrariwise, girls seem to be more satisfied in the support they received from teachers. These results could reflect, as in previous research, cultural or contextual differences relating to the specific classes or schools in which the research took place.

However, further analysis is needed to overcome some limitations of this research, in particular, the use of a probabilistic sampling to include a wider geographical area in Italy and different age groups than in our survey. Despite this, our explorative study might be considered a first step in the assessment of dimensions that might contribute to the promotion of adolescents’ well-being in developing programs of prevention.

## 5. Conclusions

The results of this study confirm, as in previous research, that psychological well-being and self-efficacy are positively correlated [40,42,43]. Specifically, in this research it was possible to highlight, with structural equation models, a link between self-efficacy in self-regulation of learning and well-being associated with the basic psychological needs. These results allow us to hypothesize specific paths aimed at promoting well-being at school, starting from the development of self-efficacy in self-regulation of learning. Developing positive self-efficacy beliefs could help adolescent students develop well-being. Informing students about socio-cognitive theory and self-efficacy for self-regulated learning would stimulate awareness and control about basic needs at school and psychological well-being [40]. The findings of this study have many practical implications. Students, teachers, and educators might benefit from understanding students’ self-efficacy for self-regulated learning beliefs. Students with poor knowledge of their beliefs about their abilities in self-regulation could participate to specific programs [61] to develop their beliefs and their abilities in self-regulated learning, using new strategies to promote their knowledge and their skills in self-regulated learning. The results of this study should encourage students and teachers to support adolescents’ basic psychological needs at school and to build school-related social support according to these need [23]. Furthermore, the ASBPNSS [25] could be considered an instrument that might monitor and support the relationship between basic psychological needs at school and self-efficacy. In conclusion, this study is of great significance, contributing to our understanding of how self-efficacy affects psychological well-being in adolescence. 

## Figures and Tables

**Table 1:** Fit indices for measurement models and invariance tests.

Models		χ^2^	df	Scf	CFI	TLI	RMSEA
Models based on 15 items	CFA-M15	346.070	87	1.16	0.889	0.866	0.087
	ESEM -M15	183.425	63	1.03	0.949	0.914	0.070
Models based on 11 items *	CFA-M11	102.369	41	1.19	0.966	0.954	0.062
	ESEM-M11	54.205	25	1.12	0.984	0.964	0.054
Gender invariance-M11	Configural	142.454	82	1.17	0.967	0.955	0.061
	Weak	151.576	90	1.16	0.966	0.958	0.059
	Strong	173.380	98	1.15	0.958	0.953	0.062
	Uniquenesses	181.687	109	1.16	0.960	0.959	0.058
	Variances/Covariances	185.850	115	1.16	0.961	0.962	0.056
	Means	198.728	118	1.25	0.955	0.958	0.059

Note. * Items eliminated due to no saturation on the a priori factor (items 10_14 and 15_15) or double saturations (items 2_4 and 5_13). Scf = scaling correction factor for models using FIML to deal with missing values. χ^2^ values are all statistically significant for *p* < 0.001

**Table 2:** Factor loadings and reliability indices of the ASBPNSS 11-item models.

	CFA-M11				ESEM-M11			
	Autonomy	Relatedness	Competence	Uniquenesses	Autonomy	Relatedness	Competence	Uniquenesses
Item 1_1	0.725			0.475	0.800	−0.036	−0.069	0.448
Item 3_7	0.646			0.281	0.555	0.014	0.125	0.284
Item 4_10	0.930			0.468	0.904	0.007	0.025	0.360
Item 6_2		0.848		0.269	0.044	0.790	0.051	0.271
Item 7_5		0.855		0.471	0.034	0.781	0.084	0.485
Item 8_8		0.865		0.583	−0.040	0.796	0.131	0.579
Item 9_11		0.871		0.251	0.048	0.846	0.013	0.253
Item 11_3			0.729	0.582	−0.074	−0.131	0.920	0.605
Item 12_6			0.727	0.135	−0.095	0.120	0.694	0.148
Item 13_9			0.647	0.241	0.089	0.264	0.364	0.228
Item 14_12			0.578	0.666	0.060	−0.210	0.709	0.595
Omega	0.93	0.99	0.98		0.92	0.97	0.92	

**Table 3:** Latent correlations of CFA and ESEM of the 11-item version of the ASBPNSS.

		CFA			ESEM	
	Autonomy	Relatedness	Competence	Autonomy	Relatedness	Competence
Autonomy	1			1		
Relatedness	0.532	1		0.481	1	
Competence	0.635	0.704	1	0.632	0.631	1

Note: all correlations were significant for *p* < 0.001.

**Table 4:** Fit indices and gender invariance tests of the predictive model.

Models	χ^2^	df	Scf	CFI	TLI	RMSEA
Configural	599.784	362	1.08	0.929	0.918	0.058
Weak	617.893	379	1.08	0.929	0.921	0.056
Strong	671.118	396	1.07	0.918	0.913	0.059
Uniquenesses	697.609	417	1.07	0.917	0.916	0.058
Variances/covariances	728.018	429	1.08	0.911	0.913	0.059
Means	743.085	433	1.08	0.908	0.911	0.060

## Data Availability

Research data are available by writing to the author.
